# Associations of Weight-Adjusted Body Fat and Fat Distribution with Bone Mineral Density in Chinese Children Aged 6–10 Years

**DOI:** 10.3390/ijerph17051763

**Published:** 2020-03-09

**Authors:** Jingjing Liang, Yongxin Chen, Jiahua Zhang, Bingjie Ma, Yan Hu, Yi Liu, Suifang Lin, Zheqing Zhang, Yanyan Song

**Affiliations:** 1 Department of Child Health Care, Guangzhou Women and Children’s Medical Center, Guangzhou Medical University, Guangzhou 510623, China; stliangjingjing@126.com (J.L.);; 2 Department of Nutrition and Food Hygiene, Guangdong Provincial Key Laboratory of Tropical Disease Research, School of Public Health, Southern Medical University, Guangzhou 510515, China

**Keywords:** BMD, children, fat distribution, android fat, gynoid fat

## Abstract

Although obesity is considered osteoprotective, the effects of body fat and fat distribution on bone tissue after adjusting for the effects of body weight remain uncertain. This study evaluated the relationships between fat mass, fat distribution, and bone mineral status beyond its weight-bearing effect. We recruited 466 children aged 6–10 years in China. Dual-energy X-ray absorptiometry was used to determine the bone mineral density (BMD) and bone mineral content (BMC) in the total body and total body less head (TBLH), as well as the fat mass (FM) and percentage fat mass (%FM) of the total and segmental body. Weight-adjusted measures of FM and %FM were derived using the residual method. After adjusting for the effects of covariates, we observed statistically significant, dose-dependent negative relationships between the TBLH·BMD/BMC and various weight-adjusted measures of body fat (*p* for trend: <0.001–0.038). For each standard deviation increment in the weight-adjusted total body, TBLH, trunk and limbs, the size-adjusted BMC decreased approximately 9.44, 9.28, 8.13, and 6.65 g in boys, respectively, and by approximately 13.74, 13.71, 7.84, and 12.95 g in girls, respectively. Significant inverse associations between FM accumulation in the total body and most body parts with the BMD/BMC were observed in both boys and girls after adjusting for weight and potential confounders.

## 1. Introduction

Traditionally, obesity is considered osteoprotective because of the weight-bearing effect of excess adipose tissue on the skeleton and the stimulation of osteoblast differentiation by increased mechanical stress on the bone [1,2,3]. Recently, however, this widely held belief has been challenged [4,5,6]. Fat mass (FM) tissue is metabolically active, and therefore its influence on the skeleton may also involve other non-weight-bearing effects [7,8]. Specifically, the pathophysiological role of adipose tissue in skeletal homeostasis may also involve the production of several adipokines and hormones that modulate bone remodeling. On the one hand, fat tissue is a major source of aromatase, an enzyme that converts androgen precursors to estrogens, hormones that play a pivotal protective role against osteoporosis [9]. On the other hand, adipocytes and osteoblasts share a common mesenchymal ancestor. Therefore, obesity may increase adipocyte differentiation and fat accumulation, while decreasing osteoblast differentiation and bone formation [10]. Studies have demonstrated that adipocytes release fatty acids and adipokines such as leptin and proinflammatory cytokines that are toxic and block the osteoblast differentiation pathway [7,11,12,13]. Therefore, an evaluation of the effects of FM on bone mass after excluding the weight-bearing effects will provide more reasonable insights into this relationship.

Childhood and adolescence are crucial periods for bone growth, and the bone tissue accumulated during this period accounts for about approximately half of the bone mass in adulthood [14]. Therefore, maximizing the peak bone mass may protect against osteoporotic fracture in later life [15]. Moreover, childhood obesity has reached an unprecedented epidemic level [16], and it is important to understand the relationship between fat mass and bone mass in growing children. However, the available evidence is not completely understood, and the results of published studies have been mixed. Some studies have reported a positive association between adipose tissue and childhood bone mineral density (BMD) or bone mineral content (BMC) [17,18,19,20], whereas others have reported a negative [21,22,23] or null [24,25] association.

Additionally, not all adipose tissue is equivalent [26], and the effects of FM on BMD may differ by sex and age [27]. Current consensus states that abdominal fat accumulation (i.e., apple-shaped, android), confers an increased risk of the metabolic complications of obesity, whereas gluteofemoral fat deposition (i.e., gynoid) is associated with a decreased risk of obesity-related health problems [28]. In the early 1990s, Heiss et al. reported an association between body fat distribution and BMD, and observed a higher BMD in subjects with android fat distribution [29]. A cross-sectional study found that the FM (or %FM) was inversely associated with BMD beyond the weight-bearing effect of the former, and abdominal fat in women and limb fat in men seemed to have the greatest effect on BMD, respectively [8]. The results of a prospective cohort study also revealed a negative association of diaphyseal strength at the radius with visceral adipose tissue in girls and central adiposity in boys [30]. Therefore, in addition to the total fat and lean mass, the site-specific determination of regional fat and lean mass has promising clinical value for the assessment of bone health [31].

This cross-sectional study aimed to explore the associations of body fat and fat distribution with the BMD of the total and subtotal body in Chinese children aged 6–9 years after adjusting the analysis for the effects of weight and other covariates.

## 2. Subjects and Methods

### 2.1. Subjects and Study Design

The present study recruited a total of 466 children (266 boys and 200 girls) aged 6–10 years in Guangzhou, an urban Chinese city, between December 2015 and March 2017. The subject selection process used in this study was described thoroughly in a previous publication [32]. Recruitment was performed using two methods. First, invitation letters that included the detailed inclusion and exclusion criteria were sent to several primary schools. Of the 1394 primary school children reached, 315 responded and agreed to participate in the study. Second, advertisements and referrals were used to recruit another 206 children, for a total of 521 respondents. Fifty-five of these subjects were excluded for the following reasons: twin status (n = 12); pre-term birth (n = 22); a history of a serious medical condition (n = 12); or unavailable core data (n = 9). The study was approved by the ethics committee of the School of Public Health at Sun Yat-sen University (No. 201549). Written informed consent was obtained from the parent or legal guardian of each participant prior to enrollment.

### 2.2. Data Collection

#### 2.2.1. Anthropometric and Bone Mineral Status Measurements

Height was measured to the nearest 0.1 cm using a standard stadiometer while the child was barefoot and standing in an upright position. A whole-body dual-energy X-ray absorptiometry (DXA) scan was performed for all subjects using a Discovery W device (Hologic Inc., Waltham, MA, USA). Body mass index (BMI) was calculated as weight (in kilograms)/height (in meters) squared. The calculation of z-score for BMI was based on the WHO Reference 2007 for the assessment of nutritional status for a specific age and gender. The BMC and BMD of the whole body and the total body less head (TBLH) were obtained. TBLH measures are considered more reproducible and are preferred for pediatric evaluations of bone health, as the variation in this parameter during skeletal development is lower than the femoral neck or lumbar spine measurements commonly used in adults [33]. The FM and %FM of the whole body, android region, gynoid region, trunk region, and limb region were obtained from the whole-body DXA scans. Each measurement was taken by the same well-trained operator. The coefficients of variation between 2 consecutive measurements with repositioning among 33 children on the same day were 1.09%, 1.58%, 1.7%, 3.8%, 2.5%, 3.9%, and 3.4% for the total body BMD, BMC, and total body, trunk, limb, android, and gynoid FM, respectively.

#### 2.2.2. Dietary Intakes and Physical Activity Assessment

Trained interviewers used face-to-face interviews to confirm the participants’ eligibility and collect essential information about lifestyle and dietary factors. Both the parents and the children were asked to respond to the questions together. A quantitative food frequency questionnaire (FFQ) was administered to assess the dietary intakes over the past year. To enable estimations of the amounts of food consumed, participants were shown food photographs depicting a range of portion sizes. The dietary intakes of energy, protein, fat, calcium, and magnesium were calculated using the 2009 China Food Composition Table [34]. The type and duration of each type of physical activity during a 3-day period (two weekdays and one weekend day) were recorded prospectively using a physical activity questionnaire [35]. The physical activity levels were calculated by combining the metabolic equivalent score (MET, kcal·kg^−1^·h^−1^) of each type of physical activity after multiplying it by its duration per day (h/d) [36]. Other information such as paternal BMI, maternal BMI, modes of delivery, calcium supplementation, and multi-vitamins supplementation during the last year were also collected.

### 2.3. Statistical Analysis

The sample size was estimated based on a previous study [37], with the intention of achieving a power of 80% and an alpha of 5%. A sample of 213 was required to detect a difference of 50 g (SD = 110 g) in BMC between children in the top and bottom %FM tertiles. The actual samples of boys and girls respectively provided 78% and 88% power to detect such a difference. All analyses were performed separately for boys and girls. For each group, the continuous variables are presented as means ± standard deviations, if the variable is not normally distributed, it is represented by the median and the interval between quantiles, and the categorical variables are expressed as percentages. Weight-adjusted (WA-) indices of FM and %FM were calculated using the following procedures: (1) first, we generated a regression of each FM and %FM indices on body weight and saved the residuals, which represented the non-weight-bearing effects of the FM and %FM indices. (2) We then added the residuals to the predicted values of FM and %FM at the mean body weight to yield the weight-adjusted (WA-) indices of FM and %FM [38]. We performed an analysis of covariance (ANCOVA) to compare the mean differences among the tertiles of weight-adjusted indices of FM and %FM. A multiple linear regression analysis was used to test for a linear trend in the relationships between BMD and WA-indices of FM after transformation into a standard normal Z-score by sex. The following covariates were used in both analyses: age, weight (except for weight analysis), height, physical activity, daily energy intake, energy intake adjusted (EA-)protein intake, EA-fat intake, EA-calcium intake, EA-magnesium intake, paternal BMI, maternal BMI, modes of delivery, calcium supplementation, and multi-vitamins supplementation. The size-adjusted BMC (SA_BMC) was also used to evaluate the adiposity–bone relationship, as recommended by Wilett et al. [38]. The SA_BMCs were calculated using the following model, as described elsewhere [37]:

BMC = β_1_ × Weight + β_2_ × Height + β_3_ × BA + Constant + Residual (1)

where β_i_ is a constant and the residual was derived from the linear regression model:

SA_BMC = β_1_ × Weight_mean_ + β_2_ × Height_mean_ + β_3_ × BA_mean_ + Constant + Residual. (2)

A two-tailed *p* < 0.05 was considered to indicate statistical significance. SPSS version 20.0 (IBM Corp, Armonk, NY, USA) for Windows was used for all statistical analyses.

## 3. Results

A total of 466 participants (200 girls and 266 boys) were included in the analysis. The demographic characteristics of the study group are presented in Table 1. The mean (SD) ages of girls and boys were 8.02 (0.94) and 7.95 (0.90) years, respectively (*p* = 0.535). The FM of the whole body, android region, trunk region, and limbs tended to be higher in boys than in girls (*p* < 0.05). However, no significant sex-related differences were observed in the %FM of the whole body, android, gynoid trunk, or limb region (*p* > 0.05), although significant differences were observed in the android to gynoid ratio of %FM (*p* = 0.048) and trunk to limb ratio of %FM (*p* = 0.035). No sex-related differences were observed (*p* > 0.1) in the total body and TBLH·BMD/BMC. The frequency of cesarean section was 50%. The partial correlation analysis suggested significant positive associations of most adiposity indices with the TBLH BMD (r: 0.154–0.700, *p* < 0.01) and the BMC (r: 0.097–0.682, *p* < 0.05) after adjusting for age and sex (Table S1 (Supplementary Materials)).

Table 2 and Table 3 present the associations between the WA-indices and TBLH·BMD/BMC after adjusting for the effects of covariates. Notably, statistically significant, dose-dependent negative relationships were observed between the TBLH·BMD/BMC and various weight-adjusted measures of body fat (P for trend: <0.001–0.038). For boys, the mean percentage differences in the TBLH BMD (BMC) between the highest and lowest tertiles of WA_FM in the total body, TBLH, android, gynoid, trunk, and limbs were −3.8% (−5.3%), −3.7% (−5.6%), −3.4% (−6.9%), −2.1% (−3.8%), −3.7% (−4.9%), and −3.5% (−4.6%), respectively. For girls, the corresponding differences were differences of −4.2% (−5.3%), −4.2% (−6.2%), −3.1% (−4.8%), −2.9% (−2.3%), −5.0% (−8.9%), and −3.2% (−4.0%), respectively. After these indices were converted into Z-scores per SD increment for the WA-total body, TBLH, android, gynoid, trunk, and limbs, the respective BMD (BMC) values had decreased by 11.75 × 10^−3^ (17.32), 11.53 × 10^−3^ (16.71), 9.94 × 10^−3^ (16.70), 5.05 × 10^−3^ (9.26), 11.51 × 10^−3^ (16.15), and 7.08 × 10^−3^ g/cm^2^ (10.74 g) in boys and by 15.69 × 10^−3^ (23.82), 15.60 × 10^−3^ (23.56), 13.00 × 10^−3^ (23.34), 7.57 × 10^−3^ (6.63), 14.25 × 10^−3^ (27.98), and 13.21 × 10^−3^ g/cm^2^ (18.87 g) in girls. Similar evidence supporting associations between the WA-indices and total BMD/BMC was determined after adjusting for covariates (Tables S2 and S3 (Supplementary Materials)).

The associations between adiposity measures and SA-BMC are presented in Table 4. After adjusting for body size, the relationship between most body fat indices and BMC were attenuated but remained significant. Among boys, those in the lowest WA-total body, TBLH, trunk, and limb tertiles had SA-BMC values that were 3.0%, 3.0%, 2.7%, and 2.9% higher than those in the highest tertiles, respectively. Among girls, the corresponding differences were 3.8%, 4.1%, 2.7%, and 3.5%, respectively. For each SD increment in the WA-total body, TBLH, trunk, and limbs, the SA-BMC decreased by approximately 9.44, 9.28, 8.13, and 6.65 g in boys, respectively, and by approximately 13.74, 13.71, 7.84, and 12.95 g in girls, respectively.

## 4. Discussion

In this cross-sectional study of 466 Chinese children aged 6–10 years, we observed significant inverse associations between the FM deposition in the total body and various body sites and the BMD/BMC in both sexes after adjusting for the effects of for mechanical loading and potential confounders. Our results suggest that measurements of bone health status and strategies to prevent bone loss should be clinical priorities for obese children.

The concept that a higher body mass results in greater skeletal loading and increased bone mineral accrual has been widely accepted. The results of our study further demonstrate the positive association between weight and bone mass. Consistent with our findings, the majority of previous studies involving adults [27,39,40,41,42,43] or children [44] revealed a positive correlation between FM and BMD/BMC without controlling for body weight. However, after we statistically removed the mechanical loading effect of body weight, we observed significant inverse relationships between various body fat measures and bone mineral status indices. Our results were inconsistent with those of previous studies. In a study of 20 obese pre-pubertal children and maturation-matched control subjects in France, Rocher et al. reported that obese children displayed lower whole-body BMD (0.88 versus 0.96 g/cm^2^, *p* < 0.05) and BMC values (1191 versus 1510 g, *p* < 0.01) when compared with controls after controlling for body weight and lean mass [45]. In a cross-sectional study of 60 Caucasian female subjects between 10 and 19 years of age, the percent of body fat was associated with a suboptimal attainment of peak bone mass [46]. Analogous findings were also reported from studies of adults. In a study of 7137 men, 4585 premenopausal women and 2248 postmenopausal women aged 25–64 years in China, FM was significantly inversely associated with BMC in the whole body and total hip in an analysis stratified by 5-kg body weight increments [8].

Several potential mechanisms have been proposed to explain the relationship between adipose tissue and bone. First, both osteoblasts and adipocytes share a common progenitor, the bone marrow mesenchymal stem cell [47]. Second, adipose tissue secretes various inflammatory cytokines, including interleukin (IL)-6 and tumor necrosis factor α (TNFα). The increased levels of proinflammatory cytokines in the circulation and tissues of an obese individual may promote osteoclast activity and bone resorption by modifying the receptor activator of NF-κB (RANK)/RANK ligand/osteoprotegerin pathway [13,48]. Third, the excessive secretion of leptin by adipocytes may affect bone growth by activating fibroblast growth factor 23 or regulating osteocalcin. Leptin may also activate hormones that regulate bone tissue via the hypothalamic–pituitary growth hormone axis [49]. Adiponectin also has indirect effects on bone that may be mediated via modulations of growth factor actions or insulin sensitivity [50]. Fourth, a high fat intake may interfere with intestinal calcium absorption and therefore reduce the availability of calcium for bone formation [51].

Beyond FM per se, the pattern of fat distribution (android versus gynoid or peripheral) has different effects on the production of adipokines and cytokines and, consequently, the risk of metabolic diseases [28]. The android region is the main site of visceral adipose tissue (VAT) accumulation. An elevated VAT level is associated with systemic inflammation. Previous studies have suggested that abdominal (android) fat accumulation is more closely associated with metabolic complications, whereas fat accumulation predominantly in the gluteofemoral region and leg (gynoid) is associated with a lower risk of metabolic disorder and may even be protective [28]. In a study of 8833 adults aged 18–64.9 years, Zhang et al. observed that excess VAT in the intra-abdominal region may have a negative pathophysiologic influence on bone [52]. In our study, however, modest inverse correlations of the WA-android FM/FM% and WA-gynoid FM/FM% with the TBLH·BMD were detected for both sexes after adjusting for the effects of mechanical loading and other potential confounders. Previous studies have proven that aging induces a reallocation of FM at the organismal level from subcutaneous to visceral adipose depots, while cellular senescence impairs the capacity of adipocyte precursors to regenerate and differentiate. Ultimately, these changes lead to a loss of function in the adipose tissue [53,54]. The relatively normal functions of adipocytes in children might explain the modest adverse correlation between fat distribution and bone mass.

Our study has several strengths. Most notably, to our knowledge, this is the first report of the non-weight-bearing-related associations of body fat and fat distribution with BMD and BMC in pre-pubertal children. There are some limitations to this study. One limitation concerns the narrow age range of the participating children (6–10 years). Consequently, our results cannot be generalized to other age groups, as the results would differ between children and adolescents because of the effects of increased growth hormone and sex steroid levels on BMD during puberty [55]. The future studies should explore these relationships in different age groups. Second, the cross-sectional observational study design led to an inability to demonstrate causality. Third, we did not adjust for the intake of vitamin D because of a lack of database of food vitamin D content in China. Moreover, some unmeasured variables may have led to residual confounding. Finally, we applied data derived from a questionnaire to estimate the dietary consumption and physical activity levels, and therefore the data might be subject to recall bias.

## 5. Conclusions

Our research demonstrated that body FM has a negative effect on the BMD/BMC in both boys and girls. Our findings highlight the importance of bone testing and strategies to improve bone health in obese children, and provide a rationale for the further exploration of the mechanisms underlying this observed relationship.

## Figures and Tables

**Table 1:** Characteristics of the study participants.

	MEAN ± SD/Median (P75–P25)/N (%)		
Variables	Boys (N = 266)	Girls (N = 200)	*p*
Age (y)	7.95 ± 0.90	8.02 ± 0.94	0.535
Weight (Kg)	27.28 ± 7.84	25.06 ± 5.50	<0.001
Height (cm)	128.9 ± 8.3	128.5 ± 7.8	0.656
BMI (kg/m^2^)	15.5 (2.95)	14.7 (2.30)	<0.001
BMI-Zscore	−0.031 ± 1.49	−0.505 ± 1.17	<0.001
Fat indices			
Total fat (Kg)	6.09 (3.74)	6.63 (3.17)	<0.001
Total fat% (%)	24.42 (8.92)	28.27 (8.29)	0.134
TBLH fat (Kg)	5.22 (3.75)	5.86 (3.11)	<0.001
TBLH t fat% (%)	24.66 (10.21)	29.16 (9.63)	0.149
Android fat (Kg)	0.31 (0.26)	0.32 (0.19)	<0.001
Android fat% (%)	21.54 (10.34)	23.78 (9.06)	0.066
Gynoid fat (Kg)	1.05 (0.72)	1.21 (0.60)	0.001
Gynoid fat% (%)	30.78 (9.04)	35.22 (8.57)	0.162
Android to Gynoid %FM ratio	0.73 (0.17)	0.71 (0.15)	0.048
Trunk fat (Kg)	2.17 (1.46)	2.34 (1.21)	0.001
Trunk fat% (%)	21.02 (8.38)	24.03 (8.64)	0.349
Limb fat (Kg)	3.11 (2.23)	3.48 (1.75)	<0.001
Limb fat% (%)	28.30 (11.0)	34.03 (10.0)	0.081
Trunk to Limb %FM ratio	0.32 (0.16)	0.39 (0.16)	0.035
TBLH bone mineral density (g/cm^2^)	0.613 ± 0.07	0.602 ± 0.06	0.12
TBLH bone mineral content (g)	583 ± 114	585 ± 106	0.278
Total bone mineral density (g/cm^2^)	0.791 ± 0.06	0.767 ± 0.06	0.431
Total bone mineral content (g)	936 ± 139	915 ± 133	0.335
Energy intake (Kcal)	1422 (554)	1267 (537)	0.228
Physical activity (MET.h/d)	40.0 (4.4)	38.2 (4.6)	0.05
EA-Protein intake (g)	67.13 ± 9.66	61.68 ± 9.47	0.991
EA-Fat intake (g)	46.52 ± 10.87	41.10 ± 8.80	0.197
EA-Calcium intake (mg)	508 (166)	477 (152)	0.244
EA-Magnesium intake (mg)	262 (51)	251 (41)	0.59
Paternal BMI	23.5 (3.9)	23.7 (3.3)	0.911
Maternal BMI	21.5 (3.4)	21.0 (3.3)	0.212
Calcium supplementation			0.213
yes	115 (43.2)	75 (37.5)	
no	151 (56.8)	125 (62.5)	
Multi-vitamins supplementation			0.821
yes	46 (17.3)	33 (16.5)	
no	220 (82.7)	167 (83.5)	

BMI: body mass index; FM: fat mass; %FM: the percentage of fat mass; BMD: bone mineral density; BMC: bone mineral content; TBLH: the total body less head; EA-: nutrient by energy intake adjust.

**Table 2:** Covariate-adjusted mean (SEM) TBLH BMD by tertiles of each WA-index of FM.

	TBLH BMD (g/cm^2^)						%Diff	ANCOVA		Z-Score	
	Q1		Q2		Q3			P-Diff	P-Trend	Linear Regression	
	Mean ± SEM	N	Mean ± SEM	N	Mean ± SEM	N				B	SEM
	g/cm²		g/cm²		g/cm²					10^3^g/cm²	10^3^g/cm²
**Boys**											
Weight	0.583 ± 0.005	89	0.606 ± 0.004 ^††^	89	0.655 ± 0.005 ^†††^	88	12.3	<0.001	<0.001	35.45	2.60 ***
WA-total FM	0.626 ± 0.003	89	0.618 ± 0.003 ^†^	89	0.602 ± 0.003 ^†††^	88	−3.8	<0.001	<0.001	−11.75	2.15 ***
WA-total %FM	0.623 ± 0.003	89	0.616 ± 0.003	89	0.607 ± 0.003 ^†^	88	−2.6	0.004	0.001	−6.76	2.16 **
WA-TBLH FM	0.624 ± 0.003	89	0.614 ± 0.003	89	0.601 ± 0.003 ^†††^	88	−3.7	<0.001	<0.001	−11.53	2.15 ***
WA-TBLH %FM	0.622 ± 0.003	89	0.616 ± 0.003	89	0.607 ± 0.003 ^†^	88	−2.4	0.008	0.002	−6.39	2.14 **
WA- Android FM	0.625 ± 0.003	89	0.616 ± 0.003	89	0.604 ± 0.003 ^††^	88	−3.4	0.001	<0.001	−9.94	2.01 ***
WA- Android %FM	0.620 ± 0.003	89	0.615 ± 0.003	89	0.611 ± 0.003	88	−1.5	0.114	0.038	−4.95	2.04 *
WA-Gynoid FM	0.622 ± 0.003	89	0.615 ± 0.003	89	0.609 ± 0.003	88	−2.1	0.024	0.006	−5.05	1.87 **
WA-Gynoid %FM	0.621 ± 0.003	89	0.615 ± 0.003	89	0.609 ± 0.003	88	−1.9	0.033	0.009	−4.84	2.01 *
WA-android to Gynoid %FM ratio	0.615 ± 0.003	89	0.616 ± 0.003	89	0.614 ± 0.003	88	−0.2	0.911	0.940	0.62	1.78
WA-trunk FM	0.622 ± 0.003	89	0.618 ± 0.003	89	0.599 ± 0.003 ^†††^	88	−3.7	<0.001	<0.001	−11.51	2.12 ***
WA-trunk %FM	0.622 ± 0.003	89	0.614 ± 0.003	89	0.604 ± 0.003 ^††^	88	−2.9	0.002	0.001	−5.55	1.99 **
WA-limb FM	0.626 ± 0.003	89	0.615 ± 0.003	89	0.604 ± 0.003 ^†††^	88	−3.5	<0.001	<0.001	−7.08	2.01 **
WA-limb %FM	0.623 ± 0.003	89	0.614 ± 0.003	89	0.608 ± 0.003 ^††^	88	−2.4	0.003	0.001	−5.17	2.04 *
WA-trunk to Limb %FM ratio	0.624 ± 0.003	89	0.617 ± 0.003	89	0.604 ± 0.003	88	−3.2	<0.001	<0.001	−8.22	2.26 ***
**Girls**											
Weight	0.579 ± 0.005	67	0.601 ± 0.004 ^†††^	67	0.633 ± 0.005 ^†††^	66	9.3	<0.001	<0.001	47.96	4.22 ***
WA-total FM	0.618 ± 0.003	67	0.604 ± 0.003 ^†††^	67	0.592 ± 0.004 ^†††^	66	−4.2	<0.001	<0.001	−15.69	2.34 ***
WA-total %FM	0.618 ± 0.004	67	0.603 ± 0.003 ^††^	67	0.593 ± 0.003 ^†††^	66	−4.0	<0.001	<0.001	−12.50	2.30 ***
WA-TBLH FM	0.619 ± 0.004	67	0.602 ± 0.003 ^†††^	67	0.593 ± 0.004 ^†††^	66	−4.2	<0.001	<0.001	−15.60	2.34 ***
WA-TBLH %FM	0.617 ± 0.003	67	0.600 ± 0.003 ^††^	67	0.589 ± 0.003 ^†††^	66	−4.5	<0.001	<0.001	−12.19	2.31 ***
WA- Android FM	0.615 ± 0.004	67	0.603 ± 0.003	67	0.596 ± 0.004 ^††^	66	−3.1	0.007	0.002	−13.00	2.90 ***
WA- Android %FM	0.614 ± 0.004	67	0.605 ± 0.003	67	0.595 ± 0.004 ^††^	66	−3.1	0.007	0.002	−10.28	2.33 ***
WA-Gynoid FM	0.615 ± 0.003	67	0.602 ± 0.003 ^†^	67	0.597 ± 0.004 ^†^	66	−2.9	0.004	0.001	−7.57	2.36 **
WA-Gynoid %FM	0.612 ± 0.003	67	0.604 ± 0.003	67	0.598 ± 0.004 ^†^	66	−2.3	0.033	0.009	−8.55	2.46 **
WA-android to Gynoid %FM ratio	0.611 ± 0.003	67	0.598 ± 0.003	67	0.605 ± 0.004	66	−1.0	0.039	0.273	−3.50	2.18
WA-trunk FM	0.618 ± 0.003	67	0.600 ± 0.003 ^††^	67	0.587 ± 0.003 ^†††^	66	−5.0	<0.001	<0.001	−14.25	2.06 ***
WA-trunk %FM	0.618 ± 0.003	67	0.599 ± 0.003 ^†††^	67	0.588 ± 0.003 ^†††^	66	−4.9	<0.001	<0.001	−12.73	2.08 ***
WA-limb FM	0.616 ± 0.004	67	0.602 ± 0.003 ^††^	67	0.596 ± 0.004 ^†††^	66	−3.2	<0.001	<0.001	−13.21	2.27 ***
WA-limb %FM	0.615 ± 0.004	67	0.603 ± 0.003 ^†††^	67	0.597 ± 0.004 ^†††^	66	−2.9	0.004	0.001	−10.85	2.34 ***
WA-trunk to Limb %FM ratio	0.618 ± 0.003	67	0.605 ± 0.003	67	0.591 ± 0.004	66	−4.4	<0.001	<0.001	−12.46	2.17 ***

FM: fat mass; %FM: the percentage of fat mass; BMD: bone mineral density; BMC: bone mineral content; TBLH: the total body less head; WA-: body weight adjusted; EA-: nutrient by energy intake adjusted. Analysis of covariance (ANCOVA) and linear regression were carried out, controlling for age, weight (except for weight analysis), height, physical activity, daily energy intake, EA-protein intake, EA-fat intake, EA-calcium intake, EA-magnesium intake, paternal BMI, maternal BMI, modes of delivery, calcium supplementation, and multi-vitamins supplementation. %Diff.: percentage difference = (Q3 − Q1)/Q1 × 100%. P-diff: P-diff for group difference; P-trend: P-trend for linear trend. ^†^, ^††^, ^†††^: compared with Q1, *p* < 0.05, *p* < 0.01, *p* < 0.001 (Bonferroni). *, **, ***: *p* for the linear trend (linear regression), *p* < 0.05, *p* < 0.01, *p* < 0.001.

**Table 3:** Covariate-adjusted mean (SEM) TBLH BMC by tertiles of each WA-index of FM.

	TBLH BMC (g)						%Diff	ANCOVA		Z-Score	
	Q1		Q2		Q3			P-Diff	P-Trend	Linear Regression	
	Mean ± SEM	N	Mean ± SEM	N	Mean ± SEM	N				B	SEM
	g		g		g					g	g
**Boys**											
Weight	553 ± 8.6	89	579 ± 6.1	89	629 ± 8.3 ^†††^	88	13.7	<0.001	<0.001	57.83	4.08 ***
WA-total FM	602 ± 5.3	89	588 ± 4.8	89	570 ± 5.1 ^††^	88	−5.3	0.001	<0.001	−17.32	3.41 ***
WA-total %FM	603 ± 4.9	89	589 ± 4.7	89	569 ± 4.8 ^†††^	88	−5.6	<0.001	<0.001	−15.73	3.31 ***
WA-TBLH FM	603 ± 5.3	89	588 ± 4.7	89	569 ± 5.1 ^††^	88	−5.6	0.001	<0.001	−16.71	3.41 ***
WA-TBLH %FM	602 ± 4.9	89	590 ± 4.7	89	569 ± 4.8 ^†††^	88	−5.5	<0.001	<0.001	−15.23	3.29 ***
WA-Android FM	608 ± 5.1	89	588 ± 4.6	89	566 ± 5.0 ^†††^	88	−6.9	<0.001	<0.001	−16.70	3.15 ***
WA-Android %FM	601 ± 4.9	89	587 ± 4.6	89	572 ± 4.9 ^††^	88	−4.8	0.004	0.001	−15.61	3.09 ***
WA-Gynoid FM	599 ± 4.9	89	586 ± 4.7	89	576 ± 4.8	88	−3.8	0.008	0.002	−9.26	2.93 **
WA-Gynoid %FM	600 ± 4.8	89	585 ± 4.7	89	576 ± 4.8 ^†^	88	−4.0	0.003	0.001	−10.43	3.12 **
WA-android to Gynoid %FM ratio	594 ± 4.8	89	592 ± 4.7	89	575 ± 4.7	88	−3.2	0.009	0.006	−6.14	2.77 *
WA-trunk FM	596 ± 5.6	89	585 ± 4.9	89	567 ± 5.1 ^††^	88	−4.9	0.001	<0.001	−16.15	3.44 ***
WA-trunk %FM	601 ± 5.2	89	584 ± 4.7	89	563 ± 4.9 ^†††^	88	−6.3	<0.001	<0.001	−15.19	3.09 ***
WA-limb FM	603 ± 5.0	89	582 ± 4.7	89	575 ± 4.9 ^†^	88	−4.6	0.001	<0.001	−10.74	3.18 **
WA-limb %FM	603 ± 4.8	89	585 ± 4.7	89	572 ± 4.8 ^††^	88	−5.1	0.001	<0.001	−11.46	3.17 ***
WA-trunk to Limb %FM ratio	605 ± 5.1	89	587 ± 4.7	89	568 ± 4.7	88	−6.1	<0.001	<0.001	−17.38	3.48 ***
**Girls**											
Weight	566 ± 8.9	67	586 ± 6.6 ^†††^	67	617 ± 8.4 ^†††^	66	9.0	0.002	0.001	65.30	6.82 ***
WA-total FM	607 ± 5.9	67	588 ± 5.4 ^†††^	67	575 ± 6.1 ^†††^	66	−5.3	0.003	0.001	−23.82	3.84 ***
WA-total %FM	609 ± 5.8	67	587 ± 5.2 ^†††^	67	573 ± 6.0 ^†††^	66	−5.9	<0.001	<0.001	−20.07	3.72 ***
WA-TBLH FM	612 ± 5.9	67	584 ± 5.3 ^††^	67	574 ± 6.1 ^†††^	66	−6.2	<0.001	<0.001	−23.56	3.84 ***
WA-TBLH %FM	609 ± 5.9	67	585 ± 5.2 ^†††^	67	575 ± 5.9 ^†††^	66	−5.6	<0.001	<0.001	−19.44	3.74 ***
WA- Android FM	606 ± 6.3	67	586 ± 5.4	67	577 ± 6.2 ^†††^	66	−4.8	0.011	0.004	−23.34	4.63 ***
WA-Android %FM	605 ± 6.2	67	589 ± 5.4	67	575 ± 5.9 ^†††^	66	−5.0	0.008	0.002	−20.47	3.67 ***
WA-Gynoid FM	598 ± 5.9	67	588 ± 5.4	67	584 ± 5.9 ^†^	66	−2.3	0.284	0.131	−6.63	3.90
WA-Gynoid %FM	600 ± 5.8	67	588 ± 5.3 ^†^	67	581 ± 5.9 ^††^	66	−3.2	0.097	0.035	−12.83	4.00 **
WA-android to Gynoid %FM ratio	604 ± 5.5	67	581 ± 5.3	67	585 ± 5.4	66	−3.1	0.007	0.015	−9.96	3.46 **
WA-trunk FM	615 ± 5.7	67	578 ± 5.3 ^†††^	67	560 ± 5.8 ^†††^	66	−8.9	<0.001	<0.001	−27.98	3.32 ***
WA-trunk %FM	613 ± 5.6	67	581 ± 5.4 ^†††^	67	560 ± 5.8 ^†††^	66	−8.6	<0.001	<0.001	−25.83	3.35 ***
WA-limb FM	604 ± 5.8	67	585 ± 5.4 ^†††^	67	580 ± 6.0 ^†††^	66	−4.0	0.015	0.008	−18.87	3.74 ***
WA-limb %FM	605 ± 5.8	67	586 ± 5.4 ^††^	67	579 ± 5.8 ^†††^	66	−4.3	0.010	0.003	−15.38	3.84 ***
WA-trunk to Limb %FM ratio	609 ± 5.7	67	590 ± 5.2	67	570 ± 6.0 ^††^	66	−6.4	<0.001	<0.001	−20.63	3.50 ***

FM: fat mass; %FM: the percentage of fat mass; BMD: bone mineral density; BMC: bone mineral content; TBLH: the total body less head; WA-: body weight adjusted; EA-: nutrient by energy intake adjusted. Analysis of covariance (ANCOVA) and linear regression were carried out, controlling for age, weight (except for weight analysis), height, physical activity, daily energy intake, EA-protein intake, EA-fat intake, EA-calcium intake, EA-magnesium intake, paternal BMI, maternal BMI, modes of delivery, calcium supplementation, and multi-vitamins supplementation. %Diff.: percentage difference = (Q3 − Q1)/Q1 × 100%. P-diff: P-diff for group difference; P-trend: P-trend for linear trend. ^†^, ^††^, ^†††^: compared with Q1, *p* < 0.05, *p* < 0.01, *p* < 0.001 (Bonferroni). *, **, ***: P for the linear trend (linear regression), *p* < 0.05, *p* < 0.01, *p* < 0.001.

**Table 4:** Covariate-adjusted mean (SEM) SA_TBLH BMC by tertiles of each WA-index of FM.

	SA_TBLH BMC (g)						%Diff	ANCOVA		Linear Regression	
	Q1		Q2		Q3			P-Diff	P-Trend		
	Mean ± SEM	N	Mean ± SEM	N	Mean ± SEM	N				B	SEM
**Boys**											
Weight	579 ± 3.7	89	582 ±2.6	89	597 ± 3.5	88	3.1	0.003	0.006	−0.88	2.25
WA-total FM	594 ± 2.9	89	588 ± 2.6	89	576 ± 2.8 ^†††^	88	−3.0	<0.001	<0.001	−9.44	1.89 ***
WA-total %FM	591 ± 2.8	89	588 ± 2.7	89	580 ± 2.7	88	−1.9	0.012	0.004	−6.22	1.87 **
WA-TBLH FM	594 ± 2.9	89	589 ± 2.6	89	576 ± 2.8 ^†††^	88	−3.0	<0.001	<0.001	−9.28	1.88 ***
WA-TBLH %FM	591 ± 2.8	89	588 ± 2.7	89	580 ± 2.7	88	−1.9	0.019	0.007	−5.99	1.86 **
WA- Android FM	592 ± 2.9	89	586 ± 2.7	89	581 ± 2.9	88	−1.9	0.055	0.016	−6.00	1.80
WA- Android %FM	590 ± 2.8	89	585 ± 2.6	89	583 ± 2.8	88	−1.2	0.238	0.107	−3.88	1.78 *
WA-Gynoid FM	591 ± 2.8	89	586 ± 2.6	89	581 ± 2.7	88	−1.7	0.081	0.025	−3.98	1.63 *
WA-Gynoid %FM	591 ± 2.7	89	588 ± 2.6	89	580 ± 2.7	88	−1.9	0.015	0.005	−5.16	1.73 **
WA-android to Gynoid %FM ratio	585 ± 2.7	89	586 ± 2.6	89	588 ± 2.6	88	0.5	0.762	0.477	1.20	1.54
WA-trunk FM	589 ± 2.9	89	586 ± 2.6	89	573 ± 2.7 ^††^	88	−2.7	<0.001	<0.001	−8.13	1.83 ***
WA-trunk %FM	588 ± 2.9	89	584 ± 2.6	89	576 ± 2.7 ^†^	88	−2.0	0.013	0.005	−3.97	1.70 *
WA-limb FM	594 ± 2.7	89	587 ± 2.6	89	577 ± 2.7 ^††^	88	−2.9	<0.001	<0.001	−6.65	1.74 ***
WA-limb %FM	593 ± 2.7	89	586 ± 2.6	89	579 ± 2.7	88	−2.4	0.003	0.001	−5.67	1.76 **
WA-trunk to Limb %FM ratio	592 ± 2.9	89	589 ± 2.6	89	578 ± 2.7	88	−2.4	0.001	0.001	−7.43	1.96 ***
**Girls**											
Weight	591 ± 4.1	67	588 ± 3.1	67	591 ± 3.9	66	0.0	0.740	0.996	−0.56	3.73
WA-total FM	602 ± 3.2	67	590 ± 2.9 ^†^	67	579 ± 3.3 ^†††^	66	−3.8	<0.001	<0.001	−13.74	2.07 ***
WA-total %FM	602 ± 3.1	67	590 ± 2.9 ^†^	67	579 ± 3.2 ^†††^	66	−3.8	<0.001	<0.001	−11.90	2.00 ***
WA-TBLH FM	604 ± 3.2	67	588 ± 2.8 ^†^	67	579 ± 3.3 ^††^	66	−4.1	<0.001	<0.001	−13.71	2.06 ***
WA-TBLH %FM	602 ± 3.2	67	589 ± 2.8 ^†^	67	580±3.2 ^†† †^	66	−3.7	<0.001	<0.001	−11.71	2.01 ***
WA- Android FM	597 ± 3.5	67	589 ± 3.0	67	585 ± 3.4	66	−2.0	0.081	0.030	−8.81	2.62
WA- Android %FM	600 ± 3.4	67	591 ± 2.9	67	581 ± 3.2	66	−3.2	0.001	0.001	−8.74	2.07 ***
WA-Gynoid FM	599 ± 3.2	67	587 ± 2.9	67	585 ± 3.1	66	−2.3	0.004	0.003	−7.45	2.07 ***
WA-Gynoid %FM	598 ± 3.1	67	591 ± 2.9	67	582 ± 3.2	66	−2.7	0.002	<0.001	−8.94	2.14 ***
WA-android to Gynoid %FM ratio	594 ± 3.0	67	585 ± 3.0	67	592 ± 3.0	66	−0.3	0.074	0.689	−1.92	1.93
WA-trunk FM	593 ± 3.2	67	583 ± 3.0 ^†^	67	577 ± 3.2 ^††^	66	−2.7	0.002	0.001	−7.84	1.94 ***
WA-trunk %FM	596 ± 3.0	67	581 ± 2.9 ^††^	67	576 ± 3.1 ^†††^	66	−3.4	<0.001	<0.001	−8.46	1.90 ***
WA-limb FM	601 ± 3.1	67	590 ± 2.9 ^†^	67	580 ± 3.2 ^†††^	66	−3.5	<0.001	<0.001	−12.95	1.96 ***
WA-limb %FM	600 ± 3.1	67	590 ± 2.9	67	582 ± 3.1 ^††^	66	−3.0	0.001	0.001	−11.32	2.02 ***
WA-trunk to Limb %FM ratio	602 ± 3.1	67	591 ± 2.8	67	578 ± 3.2	66	−4.0	<0.001	<0.001	−11.52	1.90 ***

FM: fat mass; %FM: the percentage of fat mass; BMD: bone mineral density; BMC: bone mineral content; TBLH: the total body less head; WA-: body weight adjusted; EA-: nutrient by energy intake adjusted. Analysis of covariance (ANCOVA) and linear regression were carried out, controlling for age, weight (except for weight analysis), height, physical activity, daily energy intake, EA-protein intake, EA-fat intake, EA-calcium intake, EA-magnesium intake, paternal BMI, maternal BMI, modes of delivery, calcium supplementation, and multi-vitamins supplementation. %Diff.: percentage difference = (Q3 − Q1)/Q1 × 100%. P-diff: P-diff for group difference; P-trend: P-trend for linear trend. ^†^, ^††^, ^†††^: compared with Q1, *p* < 0.05, *p* < 0.01, *p* < 0.001 (Bonferroni). *, **, ***: P for the linear trend (linear regression), *p* < 0.05, *p* < 0.01, *p* < 0.001.

## Supplementary Materials

The following are available online at https://www.mdpi.com/1660-4601/17/5/1763/s1, Table S1: Partial correlation TBLH·BMD/BMC by tertiles of each index of FM after controlling by sex and age. Table S2: Covariate-adjusted mean (SEM) Total BMD by tertiles of each WA-index of FM. Table S3: Covariate-adjusted mean (SEM) Total BMC by tertiles of each WA-index of FM.
