# Supplementary material for: Associations of Weight-Adjusted Body Fat and Fat Distribution with Bone Mineral Density in Chinese Children Aged 6–10 Years
Source: Int J Environ Res Public Health. 2020 Mar 9;17(5):1763. doi: 10.3390/ijerph17051763 (PMC7084411; doi:10.3390/ijerph17051763)
Supplement: Supplementary file 1 [file ijerph-17-01763-s001.pdf]

Table S1 Partial correlation TBLH BMD/BMC by tertiles of each index of FM after controlling by sex and age

**Variables**	**R**	**P**
Subtotal BMD		
Total fat(Kg)	0.697	<0.001
Total fat%(%)	0.482	<0.001
TBLH fat(Kg)	0.694	<0.001
TBLH fat%(%)	0.471	<0.001
Android fat(Kg)	0.631	<0.001
Android fat%(%)	0.490	<0.001
Gynoid fat(Kg)	0.656	<0.001
Gynoid fat%(%)	0.346	<0.001
Android to Gynoid %FM ratio	0.421	<0.001
Trunk fat(Kg)	0.673	<0.001
Trunk fat%(%)	0.479	<0.001
Limb fat(Kg)	0.700	<0.001
Limb fat%(%)	0.439	<0.001
Trunk to Limb %FM ratio	0.154	0.001
Subtotal BMC		
Total fat(Kg)	0.678	<0.001
Total fat%(%)	0.436	<0.001
TBLH fat(Kg)	0.674	<0.001
TBLH fat%(%)	0.424	<0.001
Android fat(Kg)	0.598	<0.001
Android fat%(%)	0.424	<0.001
Gynoid fat(Kg)	0.638	<0.001
Gynoid fat%(%)	0.307	<0.001
Android to Gynoid %FM ratio	0.357	<0.001
Trunk fat(Kg)	0.652	<0.001
Trunk fat%(%)	0.423	<0.001
Limb fat(Kg)	0.682	<0.001
Limb fat%(%)	0.404	<0.001
Trunk to Limb %FM ratio	0.097	0.038

FM: fat mass; %FM: the percentage of fat mass;

BMD: bone mineral density; BMC: bone mineral content; TBLH: the total body less head.

Table S2 Covariate-adjusted mean (SEM) Total BMD by tertiles of each WA-index of FM

	Total BMD (g/cm²)						%Diff	ANCOVA		Z-score	
	Q1		Q2		Q3			P-diff	P-trend	Linear Regression	
	Mean ±SEM g/cm²	N	Mean ±SEM g/cm²	N	Mean ±SEM g/cm²	N				B 10^{-3}g/cm²	SEM 10^{-3}g/cm²
**Boys**											
Weight	0.768±0.007	89	0.784±0.005	89	0.826±0.006†††	88	7.6	<0.001	<0.001	22.02	4.01***
WA-total FM	0.810±0.005	89	0.797±0.004†	89	0.772±0.005†††	88	-4.7	<0.001	<0.001	- 19.41	3.30***
WA-total %FM	0.809±0.005	89	0.791±0.005	89	0.778±0.005†††	88	-3.8	<0.001	<0.001	- 14.20	3.27***
WA-TBLH FM	0.810±0.005	89	0.797±0.004†	89	0.772±0.005†††	88	-4.7	<0.001	<0.001	- 19.73	3.27***
WA-TBLH %FM	0.808±0.005	89	0.792±0.004	89	0.779±0.005†††	88	-3.6	<0.001	<0.001	- 13.54	3.26***
WA- Android FM	0.809±0.005	89	0.791±0.005††	89	0.779±0.005†††	88	-3.7	0.001	<0.001	- 11.12	3.19**
WA- Android %FM	0.804±0.005	89	0.788±0.005	89	0.786±0.005†	88	-2.2	0.023	0.012	- 7.80	3.15*
WA-Gynoid FM	0.806±0.005	89	0.791±0.004††	89	0.782±0.005†††	88	-3.0	0.004	0.001	- 11.58	2.83***
WA-Gynoid %FM	0.805±0.005	89	0.794±0.004	89	0.780±0.005†††	88	-3.1	0.002	<0.001	- 11.58	3.05***
WA-android to Gynoid %FM ratio	0.790±0.005	89	0.789±0.005	89	0.800±0.005	88	1.3	0.166	0.115	3.69	2.73
WA-trunk FM	0.806±0.005	89	0.798±0.004	89	0.768±0.005†††	88	-4.7	<0.001	<0.001	- 17.71	3.20***
WA-trunk %FM	0.807±0.005	89	0.789±0.004†	89	0.775±0.005†††	88	-4.0	<0.001	<0.001	- 12.39	2.96***
WA-limb FM	0.812±0.005	89	0.796±0.004†	89	0.771±0.005†††	88	-5.0	<0.001	<0.001	- 14.71	3.05***
WA-limb %FM	0.806±0.005	89	0.795±0.004	89	0.778±0.005†††	88	-3.5	<0.001	<0.001	- 11.76	3.11***
WA-trunk to Limb %FM ratio	0.810±0.005	89	0.794±0.005	89	0.775±0.005	88	-4.3	<0.001	<0.001	- 15.96	3.44***
**Girls**											
Weight	0.746±0.007	67	0.771±0.005†††	67	0.789±0.007†††	66	5.8	0.002	<0.001	38.95	6.11***
WA-total FM	0.783±0.005	67	0.772±0.005††	67	0.750±0.005†††	66	-4.2	0.001	<0.001	- 19.63	3.50***
WA-total %FM	0.786±0.005	67	0.768±0.005††	67	0.752±0.005†††	66	-4.3	<0.001	<0.001	- 16.87	3.36***
WA-TBLH FM	0.789±0.005	67	0.767±0.005††	67	0.751±0.005†††	66	-4.8	<0.001	<0.001	- 19.98	3.47***
WA-TBLH %FM	0.786±0.005	67	0.767±0.005††	67	0.752±0.005†††	66	-4.3	<0.001	<0.001	- 16.32	3.39***
WA- Android FM	0.778±0.006	67	0.769±0.005	67	0.759±0.006	66	-2.4	0.093	0.029	- 15.08	4.29**
WA- Android %FM	0.780±0.006	67	0.770±0.005	67	0.756±0.005†	66	-3.1	0.018	0.006	- 11.62	3.46*
WA-Gynoid FM	0.782±0.005	67	0.764±0.005	67	0.760±0.005	66	-2.8	0.010	0.006	- 11.00	3.43**
WA-Gynoid %FM	0.779±0.005	67	0.767±0.005†	67	0.759±0.005†	66	-2.6	0.032	0.010	- 12.62	3.56**
WA-android to Gynoid %FM ratio	0.773±0.005	67	0.765±0.005	67	0.769±0.005	66	-0.5	0.546	0.579	- 1.36	3.17
WA-trunk FM	0.786±0.005	67	0.766±0.005††	67	0.748±0.005†††	66	-4.8	<0.001	<0.001	- 17.63	3.01***
WA-trunk %FM	0.787±0.005	67	0.763±0.005††	67	0.749±0.005†††	66	-4.8	<0.001	<0.001	- 15.86	3.02***
WA-limb FM	0.782±0.005	67	0.769±0.005†	67	0.755±0.005†††	66	-3.5	0.004	0.001	- 16.97	3.35***
WA-limb %FM	0.780±0.005	67	0.769±0.005†	67	0.757±0.005††	66	-2.9	0.014	0.003	- 14.91	3.41***
WA-trunk to Limb %FM ratio	0.785±0.005	67	0.772±0.005	67	0.749±0.005	66	-4.6	<0.001	<0.001	- 16.62	3.19***

FM: fat mass; %FM: the percentage of fat mass; BMD: bone mineral density; BMC: bone mineral content; TBLH: the total body less head; WA-: body weight adjusted; EA-: nutrient by energy intake adjusted.

Analysis of covariance (ANCOVA) and linear regression were carried out, controlling for age, weight (except for weight analysis), height, physical activity, daily energy intake, EA-protein intake, EA-fat intake, EA-calcium intake, EA-magnesium intake, paternal BMI, maternal BMI, modes of delivery, calcium supplementation and multi-vitamins supplementation

% Diff.: percentage difference=(Q3–Q1)/Q1×100%. P-diff: P-diff for group difference; P-trend: P-trend for linear trend.

†, ††, †††: compared with Q1, P<0.05, P<0.01, P<0.001 (Bonferroni). *, **, ***: P for the linear trend (linear regression), P<0.05, P<0.01, P<0.001.

Table S3 Covariate-adjusted mean (SEM) Total BMC by tertiles of each WA-index of FM

	Total BMC(g)						%Diff	ANCOVA		Z-score	
	Q1		Q2		Q3			P-diff	P-trend	Linear Regression	
	Mean ±SEM g	N	Mean ±SEM g	N	Mean ±SEM g	N				B g	SEM g
**Boys**											
Weight	904±11.6	89	933±8.2	89	989±11.1††	88	9.4	0.001	<0.001	52.73	6.44***
WA-total FM	972±8.2	89	946±7.4†	89	909±7.9†††	88	-6.5	<0.001	<0.001	- 32.05	5.27***
WA-total %FM	974±7.7	89	941±7.3	89	912±7.4†††	88	-6.4	<0.001	<0.001	- 27.91	5,16***
WA-TBLH FM	973±8.2	89	945±7.3†	89	909±7.9†††	88	-6.6	<0.001	<0.001	- 32.38	5.24***
WA-TBLH %FM	972±7.7	89	943±7.3	89	912±7.4†††	88	-6.2	<0.001	<0.001	- 26.78	5.14***
WA- Android FM	977±8.0	89	941±7.3††	89	910±7.9†††	88	-6.9	<0.001	<0.001	- 24.45	5.01***
WA- Android %FM	968±7.7	89	938±7.2†	89	921±7.7†††	88	-4.9	0.001	<0.001	- 21.99	4.93***
WA-Gynoid FM	965±7.7	89	939±7.3	89	922±7.6†††	88	-4.5	0.001	<0.001	- 16.48	4.59***
WA-Gynoid %FM	964±7.5	89	940±7.3†	89	922±7.5†††	88	-4.4	0.001	<0.001	- 19.66	4.88***
WA-android to Gynoid %FM ratio	949±7.7	89	941±7.5	89	938±7.5	88	-1.2	0.586	0.320	- 3.56	4.40
WA-trunk FM	963±8.4	89	943±7.4	89	903±7.8†††	88	-6.2	<0.001	<0.001	- 30.41	5.21***
WA-trunk %FM	971±8.0	89	935±7.3††	89	903±7.6†††	88	-7.0	<0.001	<0.001	- 27.80	4.70***
WA-limb FM	975±7.7	89	940±7.2†	89	912±7.6††	88	-6.5	<0.001	<0.001	- 23.12	4.91***
WA-limb %FM	968±7.5	89	943±7.4	89	916±7.5†††	88	-5.4	<0.001	<0.001	- 21.09	4.96***
WA-trunk to Limb %FM ratio	976±7.9	89	941±7.3	89	910±7.4	88	-6.8	<0.001	<0.001	- 30.59	5.42***
**Girls**											
Weight	893±12.7	67	926±9.5†††	67	944±12.1†††	66	5.7	0.052	0.017	67.93	10.67***
WA-total FM	945±9.3	67	924±8.4†††	67	895±9.6††	66	-5.3	0.003	0.001	- 35.52	6.07***
WA-total %FM	951±9.2	67	917±8.2†††	67	895±9.4†††	66	-5.9	0.001	<0.001	- 30.55	5.84***
WA-TBLH FM	956±9.2	67	914±8.2†††	67	894±9.5†††	66	-6.5	0.001	<0.001	- 36.07	6.02***
WA-TBLH %FM	951±8.6	67	916±8.2†††	67	897±9.2†††	66	-5.7	0.001	<0.001	- 29.32	5.88***
WA- Android FM	943±9.9	67	917±8.6	67	903±9.8††	66	-4.2	0.038	0.012	- 33.21	7.32**
WA- Android %FM	943±9.8	67	921±8.5	67	899±9.3†††	66	-4.7	0.015	0.004	- 26.38	5.89***
WA-Gynoid FM	937±9.2	67	915±8.4	67	912±9.2	66	-2.7	0.122	0.066	- 14.40	6.05*
WA-Gynoid %FM	938±9.0	67	917±8.3††	67	909±9.3††	66	-3.1	0.077	0.032	- 20.88	6.23***
WA-android to Gynoid %FM ratio	936±8.7	67	914±8.4	67	914±8.5	66	-2.4	0.120	0.078	- 8.78	5.50
WA-trunk FM	962±8.7	67	908±8.2†††	67	876±8.8†††	66	-8.9	<0.001	<0.001	- 42.24	5.13***
WA-trunk %FM	959±8.5	67	910±8.3†††	67	877±8.8†††	66	-8.6	<0.001	<0.001	- 37.95	5.22***
WA-limb FM	943±9.1	67	919±8.4††	67	902±9.3†††	66	-4.3	0.015	0.004	- 29.18	5.85***
WA-limb %FM	942±9.1	67	919±8.4††	67	903±9.1†††	66	-4.1	0.018	0.005	- 24.26	5.99***
WA-trunk to Limb %FM ratio	950±9.0	67	924±8.2	67	890±9.4†	66	-6.3	<0.001	<0.001	- 31.19	5.50**

FM: fat mass; %FM: the percentage of fat mass; BMD: bone mineral density; BMC: bone mineral content; TBLH: the total body less head; WA-: body weight adjusted; EA-: nutrient by energy intake adjusted.

Analysis of covariance (ANCOVA) and linear regression were carried out, controlling for age, weight (except for weight analysis), height, physical activity, daily energy intake, EA-protein intake, EA-fat intake, EA-calcium intake, EA-magnesium intake, paternal BMI, maternal BMI, modes of delivery, calcium supplementation and multi-vitamins supplementation

% Diff.: percentage difference=(Q3–Q1)/Q1×100%. P-diff: P-diff for group difference; P-trend: P-trend for linear trend.

†, ††, †††: compared with Q1, P<0.05, P<0.01, P<0.001 (Bonferroni).

*, **,***: P for the linear trend (linear regression), P<0.05, P<0.01, P<0.001.
